# Effects of inoculating feruloyl esterase-producing *Lactiplantibacillus plantarum* A1 on ensiling characteristics, in vitro ruminal fermentation and microbiota of alfalfa silage

**DOI:** 10.1186/s40104-023-00837-0

**Published:** 2023-03-14

**Authors:** Fuhou Li, Samaila Usman, Wenkang Huang, Mengya Jia, Zohreh Akhavan Kharazian, Tao Ran, Fadi Li, Zitong Ding, Xusheng Guo

**Affiliations:** 1 State Key Laboratory of Herbage Improvement and Grassland Agro-Ecosystems, College of Pastoral Agriculture Science and Technology, Lanzhou University, Lanzhou, 730000 People’s Republic of China; 2 Probiotics and Biological Feed Research Centre, Lanzhou University, Lanzhou, 730000 People’s Republic of China; 3 State Key Laboratory of Herbage Improvement and Grassland Agro-Ecosystems, School of Life Sciences, Lanzhou University, No. 222 South Tianshui Road, Lanzhou, 730000 People’s Republic of China

**Keywords:** Digestibility, Ferulic acid esterase, In vitro fermentation, Rumen microbial community, Silage

## Abstract

**Background:**

Ferulic acid esterase (FAE)-secreting *Lactiplantibacillus plantarum* A1 (Lp A1) is a promising silage inoculant due to the FAE’s ability to alter the plant cell wall structure during ensiling, an action that is expected to improve forage digestibility. However, little is known regarding the impacts of Lp A1 on rumen microbiota. Our research assessed the influences of Lp A1 in comparison to a widely adopted commercial inoculant Lp MTD/1 on alfalfa’s ensilage, in vitro rumen incubation and microbiota.

**Results:**

Samples of fresh and ensiled alfalfa treated with (either Lp A1 or Lp MTD/1) or without additives (as control; CON) and ensiled for 30, 60 and 90 d were used for fermentation quality, in vitro digestibility and batch culture study. Inoculants treated silage had lower (*P* < 0.001) pH, acetic acid concentration and dry matter (DM) loss, but higher (*P* = 0.001) lactic acid concentration than the CON during ensiling. Compared to the CON and Lp MTD/1, silage treated with Lp A1 had lower (*P* < 0.001) aNDF, ADF, ADL, hemicellulose, and cellulose contents and higher (*P* < 0.001) free ferulic acid concentration. Compared silage treated with Lp MTD/1, silage treated with Lp A1 had significantly (*P* < 0.01) improved ruminal gas production and digestibility, which were equivalent to those of fresh alfalfa. Real-time PCR analysis indicated that Lp A1 inoculation improved the relative abundances of rumen’s total bacteria, fungi, *Ruminococcus albus* and *Ruminococcus flavefaciens*, while the relative abundance of methanogens was reduced by Lp MTD/1 compared with CON. Principal component analysis of rumen bacterial 16S rRNA gene amplicons showed a clear distinction between CON and inoculated treatments without noticeable distinction between Lp A1 and Lp MTD/1 treatments. Comparison analysis revealed differences in the relative abundance of some bacteria in different taxa between Lp A1 and Lp MTD/1 treatments. Silage treated with Lp A1 exhibited improved rumen fermentation characteristics due to the inoculant effects on the rumen microbial populations and bacterial community.

**Conclusions:**

Our findings suggest that silage inoculation of the FAE-producing Lp A1 could be effective in improving silage quality and digestibility, and modulating the rumen fermentation to improve feed utilization.

**Supplementary Information:**

The online version contains supplementary material available at 10.1186/s40104-023-00837-0.

## Introduction

Feed utilization efficiency is a highly important trait in ruminant production and significantly impacts the benefits of ruminant husbandry. In recent years, there has been an increasing demand for efficient utilization of forage in ruminant production because of increasing costs of grains and shortage of food supply for human nutrition [1]. Studies have shown that increasing ruminal digestibility of high-quality forage would be beneficial to the production of dairy cows, because it could increase the energy supply from volatile fatty acid (VFA) and protein from microbial protein synthesis [2, 3].

Ensiling is an effective way of preserving high quality forage. Lactic acid bacteria (LAB) inoculants are widely used in silage preparation due to their high efficiency in aiding fermentation and preventing spoilage [4, 5]. However, most inoculants currently used in silage had a major limitation of the absence of enzymatic capability to effectively degrade lignocellulose structure of forages [4]. Therefore, LAB inoculants are generally considered to have little or no impacts on nutrient digestibility of silage either in vitro or in vivo [4, 6].

Studies have proven that the extent of cell wall digestion in the rumen is largely dependent on ferulic acid, which is linked to lignin as well as polysaccharides by ether and/or ester bonds [7–9]. Therefore, silage inoculants that produce ferulic acid esterase (FAE) have been reported to have great potentials in improving the efficiency of silage digestion by secreting the FAE to breakdown the linkages between lignin and the cell wall carbohydrates of forages, and release ferulic acid from arabinoxylans during ensiling [3, 10]. Breakage of the linkage between lignin and cell wall carbohydrates facilitates further degradation of the forages in the rumen [11]. Our previous studies showed that inoculation with FAE-producing LAB during ensiling increased the permeability of plant cell wall and the accessibility of enzymes and acids to structural polysaccharides [12, 13]. The result is that FAE-producing LAB-treated silage showed lower fiber concentrations than other treatments during ensiling. Meanwhile, Li et al. [12, 14] and Usman et al. [10] showed the positive effectiveness of inoculating a FAE-producing *Lactiplantibacillus plantarum* A1 on silage enzymatic degradation in vitro. Kang et al. [15] and Jin et al. [16] have also reported an improved in situ DM and NDF digestibilities of silages treated with FAE-producing *Lentilactobacillus buchneri*. In addition, our recent research also found that inoculation of FAE-producing *L. plantarum* A1 in alfalfa silage improved the DM digestibility of dairy goat diet [17]. However, although the fiber-degrading characteristics of FAE-producing LAB have been investigated and there are several studies that confirmed the capability of FAE-producing LAB inoculants in improving the silage in vitro or in vivo DM digestibility [15–17], to date, its impacts on ruminal fermentation and microbiota are poorly understood.

Thus, the objectives of the present study were to investigate the effect of FAE-producing LAB inoculation on fermentation characteristics of alfalfa silage, in vitro feed digestion, and the major microbial groups and bacterial community involved in feed digestion and rumen fermentation, with comparison to a same type of inoculant that is widely used in silage.

## Materials and methods

The whole animal-handing protocols were reviewed and approved by Animal Ethics Committee of Lanzhou University (file No. 2010–1 and 2010–2), following the Chinese Standards for the Use and Care of Research Animals, and confirming that all experiments were performed in accordance with relevant guidelines and regulations.

### Inoculants used

In the current study, *L. plantarum* MTD/1 is a widely adopted commercial inoculant (Ecosyl Products Ltd., Stokesley, UK), generally used to improve the fermentation quality of silages, but it does not exhibit FAE activity [13, 18]. *L. plantarum* A1 is a strain that previously isolated and screened from the ensiled grass of *Elymus nutans* harvested from the Qinghai-Tibet plateau, which has been proved possessing FAE activity in addition to improving silage fermentation quality in our previous study [19]. In addition, the results showed that inoculation with FAE-producing *L. plantarum* A1 during ensiling decreased the forage (including corn stalk, alfalfa, *Pennisetum sinese* and sorghum) lignocellulose concentration with a concomitant increase in free ferulic acid concentration in silage [10, 12–14]. During the silage preparation, both strains were provided in the form of freeze-dried powder.

### Alfalfa silage preparation

Four plots of first cut alfalfa (*Medicago sativa* L.) at the late bud to early bloom stage were randomly selected and harvested from an established alfalfa field in Dingxi, Gansu Province, China. The fresh alfalfa was immediately transported to the laboratory, then the harvested alfalfa was evenly spread on the laboratory floor for naturally wilting. During the wilting period, the DM content was frequently detected using a microwave oven according to the method described by Zhang et al. [20] until the DM content reached to around 30%. Thereafter, the wilted alfalfa was chopped into approximately 2-cm fragments using a paper cutter (deli8015, Deli group Co. Ltd., Ningbo, China). The chopped forages were mixed thoroughly by plot, and about 500 g from each plot was subsampled and frozen for further chemical analysis (Table 1); the rest of the chopped forages of each plot were apportioned into nine piles (about 500 g each), randomly assigned into three inoculant treatments. The treatments were: no additives (control, CON), *L. plantarum* A1 (Lp A1), and *L. plantarum* MTD/1 (Lp MTD/1), with four replicates (plots in the field) for each treatment and three ensiling periods. During ensiling, the inoculants were dissolved in 5 mL distilled water and sprayed on the chopped alfalfa to achieve a rate of 1 $\times$10^6^ colony forming units (CFU)/g fresh alfalfa (FM); meanwhile, the CON was treated with equal volume of distilled water. All the treated piles were packed into 25 cm $\times$ 35 cm vacuum-packed polyethylene plastic bags (Shanghai Yhpak Co., Ltd., Shanghai, China) that were used as laboratory silos and vacuum-sealed to a density of approximately 0.534 g/cm^3^. The silos then were stored and ensiled at 25 ± 0.2 °C for 30, 60 and 90 d.

**Table 1:** Chemical composition of fresh alfalfa

Item^1^	Mean	SD^2^
DM, g/kg FM	312	5.92
CP, g/kg DM	247	6.17
WSC, g/kg DM	62.5	1.25
aNDF, g/kg DM	296	0.86
ADF, g/kg DM	222	0.23
ADL, g/kg DM	44.9	2.14
Hemicellulose, g/kg DM	73.6	1.09
Cellulose, g/kg DM	177	2.36
Ferulic acid, mg/kg DM	6336	153

All these results were obtained based on 4 measurements (plots in the field)

^1^*DM* Dry matter, *FM* Fresh material, *CP* Crude protein, *WSC* Water-soluble carbohydrates, *aNDF* Neutral detergent fiber, assayed with a heat-stable amylase and expressed inclusive of residual ash, *ADF* Acid detergent fiber, *ADL* Acid detergent lignin

^2^*SD* Standard deviation

### Chemical analysis of fresh alfalfa and silage

Samples of fresh and ensiled alfalfa (20 g) that collected at d 30, 60 and 90 were homogenized with 180 mL distilled water in a juice extractor for 30 s, filtered through 4 layers of medical gauze, and with pH of filtrates measured immediately using a glass electrode pH meter (Orion Star^TM^ A111, Thermo Fisher Scientific, Waltham, MA, USA). Then, the filtrates were acidified with 7.14 mol/L H_2_SO_4_ to pH = 2 and filtered using 0.22-μm dialyzer. The filtrates were used to quantify the concentrations of lactic acid, acetic acid, propionic acid, and butyric acid referring to our previously published method [14]. DM content (AOAC method 943.01) of fresh and silage samples were determined according to AOAC [21]. The dried samples were ground through a 1-mm sieve and sealed in plastic sample bags pending the subsequent analysis of the fiber fractions [amylase-treated neutral detergent fiber (ADF), acid detergent lignin (ADL)], crude protein (CP), and water-soluble carbohydrates (WSC). Concentrations of aNDF and ADF were determined by digestion in neutral detergent and acid detergent, respectively, using the batch procedures outlined for an ANKOM 2000 Fiber Analyzer (ANKOM Technology Corporation, Fairport, NY, USA). During the aNDF analysis, heat stable alpha-amylase and sodium sulfite were added to eliminate starch and protein effects, respectively, while the aNDF content is inclusive of the residual ash. The concentration of ADL was determined according to the procedures as reported earlier [22]. Hemicellulose and cellulose were calculated using aNDF−ADF and ADF−ADL, respectively. The nitrogen was measured using Kjeldahl automated apparatus (K9805, Shanghai Analytical Instrument Co., Ltd, Shanghai, China) and CP was computed by multiplying the Kjeldahl nitrogen with 6.25 [13]. The colorimetric method was used to quantify the WSC concentration of samples after fully reacting with anthrone reagents [23]. The method of Zhao et al. [24] with modification was used to extract and measure ferulic acid concentration in both fresh and silage samples.

### In vitro batch culture

The in vitro batch culture was conducted by a two­step approach using sheep as ruminal fluid donor:

#### Step 1 (preliminary experiment)

In order to achieve an obvious difference among treatments, an in vitro digestibility experiment of all silage samples was conducted using a Daisy^II^ Incubator (ANKOM Technology Corporation, Fairport, NY, USA) before in vitro batch culture. Four parallel subsamples of each silo (500 ± 5 mg) and glass beads (about 8 g) were heat-sealed within fiber bags (ANKOM F57, 50 mm $\times$ 55 mm, 25 μm porosity, ANKOM Technology Corporation, Fairport, NY, USA) using an impulse sealer (SF-400, Qingdao Ausense Packing Equipment Co., Ltd., Qingdao, China). Before sample placement, empty F57 filter bags were pre-rinsed in acetone, air-dried, and then dried thoroughly in a forced-air oven at 105 °C for 5 h. Similarly, four empty fiber bags with only glass beads were used as blanks. A total of 148 fiber bags (36 silos $\times$ 4 parallel samples + 4 blanks) were prepared for in vitro digestibility determination.

Five healthy rumen-fistulated dorper sheep (18–24 months old) were used as rumen fluid donors. The sheep were fed ad libitum at 0730 and 1730 h, and the diet of sheep comprising 40% corn silage, 20% alfalfa hay, and 40% concentrate mixture (DM basis), which was formulated to include 46.7% NDF, 35.2% ADF, 16.7% CP, and 12.6 MJ/kg of digestible energy. About 620 mL of filtered mixed rumen fluid from donors was added to each incubation jar containing pre-warmed (39 °C) buffers (rumen fluid:buffers = 1:2, v/v) and samples (each containing 37 bags) under continuous flushing with CO_2_ until final lid placement [25]. About 50 mL of ruminal mixture was prepared for each fiber bag. Four prepared incubation jars were then placed into Daisy^II^ Incubator and writhed continuously for 48 h at 39 °C. Following incubation, fiber bags were collected and rinsed with cold distilled water until the water became clear. Then, the fiber bags were squeezed gently to remove excess water, dried in an oven at 65 °C for 48 h, and finally weighed to calculate DM digestibility by the difference of residues before and after digestion.

#### Step 2 (in vitro batch culture)

According to the results of step 1, three silage samples of each treatment from a suitable ensiling period (9 silage samples = 3 treatments $\times$ 3 replicates) and 3 fresh alfalfa samples were subjected to in vitro batch culture. The in vitro batch culture was carried out in 100 mL calibrated glass syringes gas production system and repeated in two separate incubations. The ground fresh alfalfa and silage samples (500 ± 5 mg) were weighed and heat-sealed within fiber bags like step 1 but with 3 replicates for each sample. Before morning feeding, fresh rumen fluid was obtained through rumen cannula from 5 dorper donor sheep. Subsequently, rumen fluid was mixed equally and squeezed through 4 layers of cheesecloth into a sterilized flask (2500 mL) that was pre-warmed in a water bath of 39 °C. The anaerobic buffer medium was prepared according to Theodorou et al. [25] and combined with the rumen fluid in a ratio of 2:1 (v/v) under anaerobic conditions. A 50 mL mixture was immediately dispensed into each incubation glass syringe containing substrates. Four syringes containing only incubation media and empty bags were used as blanks for correcting gas production. A total of 40 glass syringe gas production systems [(9 silage samples + 3 fresh alfalfa) $\times$ 3 replicates + 4 blanks] were prepared and incubated in a 39 °C-water bath for 48 h. All of syringes were affixed to a rotary shaker to ensure that the fermenters were shaken gently during fermenting. The accumulated gas production was recorded and gas samples (5 mL each) were collected using a gastight syringe at 6, 12, 24, and 48 h of incubation, respectively. Methane concentration of mixed gas was measured using a gas chromatography (SP-3420A, Shimadzu, Japan) according to Wang et al. [26]. After 48 h of incubation, fiber bags were removed from glass syringes and rinsed 4 to 6 times using cold distilled water, squeezed gently to remove excess water, placed in an oven at 65 °C for 48 h to calculate DM digestibility. Fermentation fluid of each in vitro culture was collected for measurements of VFA, ammonia nitrogen (NH_3_-N) and used for DNA extraction and subsequent microbial analysis. The rumen fluid was immediately treated by adding 250 g/L (w/v) HPO_3_ at a ratio of 1:5 (v/v, HPO_3_:rumen fluid) for further VFA analysis using a gas chromatography (SP-3420A, Shimadzu, Japan) according to the method described by Wang et al. [26]. The concentration of NH_3_-N in rumen fluid was quantified using a colorimetric method following the procedure of Broderick and Kang [27]. The remaining part of rumen fluid was stored at −80 °C for subsequent DNA analysis.

### DNA extraction, PCR amplification, and sequencing

DNA of frozen rumen liquid (about 10 mL) was extracted with the EasyPure Stool Genomic DNA Kit (TransGen Biotech, Beijing, China) following the manufacturer’s instructions. The DNA concentration and purity was checked using a Nanodrop 2000 (Thermo Fisher Scientific, Inc., Madison, WI, USA). The polymerase chain reaction (PCR) amplification of the V3-V4 region of the bacterial 16S rRNA gene was carried out applying universal primer pairs 338F (5'-ACTCCTACGGGAGGCAGCAG-3') and 806R (5'-GGACTACHVGGGTWTCTAAT-3') [28]. Amplicon was examined on a 2% (w/v) agarose gel to verify their expected bands and size. The PCR products were extracted and purified using an AxyPrep DNA Gel Extraction Kit (Axygen Biosciences, Union City, CA, USA), and the purified DNA amplicons were quantified using a Quantus™ Fluorometer (Promega, Madison, WI, USA). DNA-Seq was done on an Illumina MiSeq PE300 platform (Illumina, San Diego, CA, USA) provided by Shanghai Majorbio Bio-Pharm Technology Co., Ltd. (Shanghai, China). The sequence information reported in this study had been deposited in the NCBI database (https://www.ncbi.nlm.nih.gov/sra/PRJNA793346).

Sequences were processed, clustered and taxonomically classified with the QIIME2 software package (Quantitative Insights Into Microbial Ecology) [29]. Clustering of operational taxonomic units (OTU) with 97% similarity using UPARSE version 7.1 [30], and at the same time, the data were identified and some chimeric DNA sequences were removed. Representative sequences for each OTU were classified and analyzed by RDP Classifier version 2.2 [31] and the 16S rRNA database (V201305, Greengenes database) with a confidence threshold of 0.8. The community diversity was estimated using the four commonly used indices in the alpha diversity index (ACE, Chao1, Shannon, and Simpson). Then, principal coordinate analysis (PCoA) was used to obtain the interrelationships of bacterial community composition from four different treatment’s samples. Venn diagram was used to detected the similarities and differences of ruminal microorganisms’ OUT among treatments.

### Quantitative real-time PCR (qPCR) analysis

The qPCR was used for absolute quantification of total bacteria, protozoa, fungi, methanogens, *Ruminococcus albus*, *Ruminococcus flavefaciens* and *Fibrobacter succinogenes*. The qPCR assay for total bacteria, methanogens and the three cellulolytic bacteria [29, 32] targeted partial sequences of the 16S rRNA gene, whereas that for protozoa and fungi targeted partial sequences of the 18S rRNA gene. A full description of the primers and thermal profiles used for total bacteria, protozoa, fungi, methanogens, *R. albus*, *R. flavefaciens* and *F. succinogenes* could be seen in Table S1. The specific operation refers to the method of Shen et al. [29].

### Statistical analysis

Chemical composition of wilted alfalfa before ensiling was presented as mean ± standard deviation (SD). The dynamical results of silage fermentation parameters, concentrations of fiber components and ferulic acid during ensiling, and digestibility in step 1 of in vitro batch culture were analyzed using general linear model of Statistical Package for Social Science (SPSS 21.0, SPSS, Inc., Chicago, IL, USA) according to a 3 $\times$ 3 factorial treatment design (three treatments and three ensiling days):

$${Y}_{ij} = \mu + {T}_{i} + {D}_{j} + {(T \times D)}_{ij} + {e}_{ij}$$

where *Y*_*ij*_ represents observation; *μ* is the overall mean; *T*_*i*_ represents the effect of three treatments (*i* = 1, 2, 3); *D*_*j*_ represents the effect of three ensiling days (*j* = 1, 2, 3); *(T *$\times$*D)*_*ij*_ represents the interaction of treatments and ensiling days, and *e*_*ij*_ represents the random residual error. Significance was considered at *P* ≤ 0.05.

Data on in vitro ruminal fermentation characteristics, qPCR of the microbial population, alpha diversity (ACE, Chao1, Shannon, and Simpson) of the bacteria, and the microbial communities’ relative abundances at phylum, and genus levels were analyzed using one-way ANOVA of SPSS 21.0. The absolute quantification data of the total bacteria, protozoa, fungi, methanogens, *R. albus*, *R. flavefaciens* and *F. succinogenes* generated by qPCR data were log transformed before conducting the one-way ANOVA to improve normality. Significant means were separated using Tukey’s multiple comparison tests at *P* ≤ 0.05. RStudio (Version 1.0.136) was used to calculate Pearson correlation coefficients and generate a heatmaps to examine the correlation between the relative abundances of bacterial genera and each of the fermentation and chemical parameters. Significant correlation was considered at *P* ≤ 0.05.

## Results

### Fermentation characteristics, fiber component and ferulic acid of alfalfa silage during ensiling

The interaction of T $\times$ D influenced both pH and lactic acid (Table 2; *P* < 0.01). As expected, both inoculants decreased silage pH and improved lactic acid concentration during ensiling, but no differences were observed in pH and lactic acid concentration between the Lp MTD/1-treated silages ensiled for 30, 60 and 90 d, while for Lp A1-treated silages, a lower pH and a higher lactic acid concentration were found in 90 d silage compared with 30 and 60 d silages. Similarly, both Lp A1 and Lp MTD/1 reduced silage DM loss during ensiling (*P* < 0.001), but no difference was observed in DM loss between Lp A1-treated silages ensiled for 30, 60 and 90 d, while the DM loss increased with the extension of ensiling time in silages inoculated with Lp MTD/1.

**Table 2:** Fermentation characteristics of alfalfa silage during ensiling

Items^1^	Ensiling days, d	Treatment (T)^2^			Mean	SEM^3^	*P*-value^4^		
		CON	Lp A1	Lp MTD/1			T	D	T $\times$ D
pH	30	5.22^Aa^	4.81^Ba^	4.86^B^	4.96	0.027	< 0.001	< 0.001	0.008
	60	5.16^Aa^	4.75^Bab^	4.84^B^	4.92				
	90	4.96^Ab^	4.70^Cb^	4.80^B^	4.82				
	Average	5.11	4.76	4.83					
Lactic acid, g/kg DM	30	60.5^Cc^	86.7^Ab^	76.8^B^	74.7	1.77	< 0.001	< 0.001	0.005
	60	68.0^Cb^	103^Aa^	80.4^B^	83.8				
	90	71.3^Ca^	96.0^Aab^	79.6^B^	82.3				
	Average	66.6	95.3	78.9					
Acetic acid, g/kg DM	30	27.1^A^	25.4^AB^	24.0^B^	25.5	0.46	< 0.001	0.111	0.514
	60	27.2^A^	25.9^AB^	23.7^B^	25.6				
	90	27.0^A^	24.2^B^	23.3^B^	24.8				
	Average	27.1^A^	25.2^B^	23.7^C^					
Propionic acid, g/kg DM	30	9.71^Bb^	9.47^Bc^	11.3^Ac^	10.2	0.49	< 0.001	< 0.001	< 0.001
	60	13.8^Ca^	19.9^Aa^	16.8^Bb^	16.8				
	90	14.9^Ba^	16.1^Bb^	19.4^Aa^	16.8				
	Average	12.8	15.2	15.8					
DM loss, g/kg DM	30	75.9^A^	52.7^B^	49.2^Bc^	59.3^b^	2.36	< 0.001	0.001	0.052
	60	79.7^A^	57.4^B^	57.6^Bb^	64.9^ab^				
	90	77.3^A^	58.4^B^	65.4^Ba^	67.0^a^				
	Average	77.7^A^	56.2^B^	57.4^B^					

^A−C^Means within a row with different superscripts differ (*P* < 0.05); ^a−c^Means within a column with different superscripts differ (*P* < 0.05)

^1^DM = dry matter

^2^CON = control (no additives); Lp A1 = silage inoculated with *L. plantarum* A1; Lp MTD/1 = silage inoculated with *L. plantarum* MTD/1

^3^SEM = standard error of means

^4^ T = treatment; D = ensiling days; T $\times$ D = the interaction between treatment and ensiling days

As presented in Table 3, unlike silages treated with Lp MTD/1, the silages inoculated with Lp A1 had lower aNDF and ADF concentrations compared with CON silages regardless of the ensiling times (*P* < 0.05). There was an T $\times$ D interaction for ferulic acid (*P* < 0.001). Ferulic acid concentration was highest in Lp A1-treated silages. Although ferulic acid concentrations were higher in Lp MTD/1-treated silages than CON ensiled for 30 and 60 d (*P* < 0.05), no difference was observed between the two treatments for 90 d silages.

**Table 3:** Fiber components and ferulic acid concentration of alfalfa silage during ensiling

Items^1^	Ensiling days, d	Treatment (T)^2^			Mean	SEM^3^	*P*-value^4^		
		CON	Lp A1	Lp MTD/1			T	D	T $\times$ D
aNDF, g/kg DM	30	297^A^	278^Bb^	287^Bb^	287^b^	2.56	< 0.001	0.001	0.108
	60	300^A^	287^Ba^	302^Aa^	296^a^				
	90	295^A^	278^Bb^	296^Aab^	290^b^				
	Average	297^A^	281^B^	295^A^					
ADF, g/kg DM	30	227^Ab^	216^Bb^	222^Ab^	222^b^	1.57	< 0.001	< 0.001	0.105
	60	236^Aa^	227^Ba^	233^ABa^	232^a^				
	90	227^Ab^	214^Bb^	228^Aab^	223^b^				
	Average	230^A^	219^B^	228^A^					
ADL, g/kg DM	30	47.5^ABc^	45.6^Bb^	49.1^Ab^	47.4^c^	0.51	< 0.001	< 0.001	0.167
	60	52.4^Ba^	49.0^Ca^	53.9^Aa^	51.8^a^				
	90	50.1^Ab^	45.6^Bb^	50.6^Ab^	48.7^b^				
	Average	50.0^B^	46.7^C^	51.2^A^					
Hemicellulose, g/kg DM	30	70.1^A^	62.0^Bab^	64.4^AB^	65.5	1.52	< 0.001	0.123	0.024
	60	63.8^AB^	60.0^Bb^	68.9^A^	64.2				
	90	68.1^AB^	64.0^Ba^	68.5^A^	66.8				
	Average	67.3^A^	62.0^B^	67.2^A^					
Cellulose, g/kg DM	30	180^Aab^	170^Bb^	173^Bb^	174	1.17	< 0.001	< 0.001	0.005
	60	183^a^	180^a^	181^a^	182				
	90	176^Ab^	168^Bb^	177^Aab^	174				
	Average	180	173	177					
Ferulic acid, mg/kg DM	30	4900^Ca^	6285^Aa^	5399^Ba^	5528	61.9	< 0.001	< 0.001	< 0.001
	60	4404^Cb^	5340^Ab^	4821^Bb^	4855				
	90	4316^Bb^	4836^Ac^	4124^Bc^	4425				
	Average	4540	5487	4781					

^A−C^Means within a row with different superscripts differ (*P* < 0.05); ^a−c^Means within a column with different superscripts differ (*P* < 0.05)

^1^DM = dry matter; aNDF = neutral detergent fiber, assayed with a heat-stable amylase and expressed inclusive of residual ash; ADF = acid detergent fiber; ADL = acid detergent lignin

^2^CON = control (no additives); Lp A1 = silage inoculated with *L. plantarum* A1; Lp MTD/1 = silage inoculated with *L. plantarum* MTD/1

^3^SEM = standard error of means

^4^ T = treatment; D = ensiling days; T $\times$ D = the interaction between treatment and ensiling days

### Effects of Lp A1 inoculation in silage on rumen fermentation characteristics and the correlations of rumen fermentation indicators with silage fiber components

**Fig. 1:**
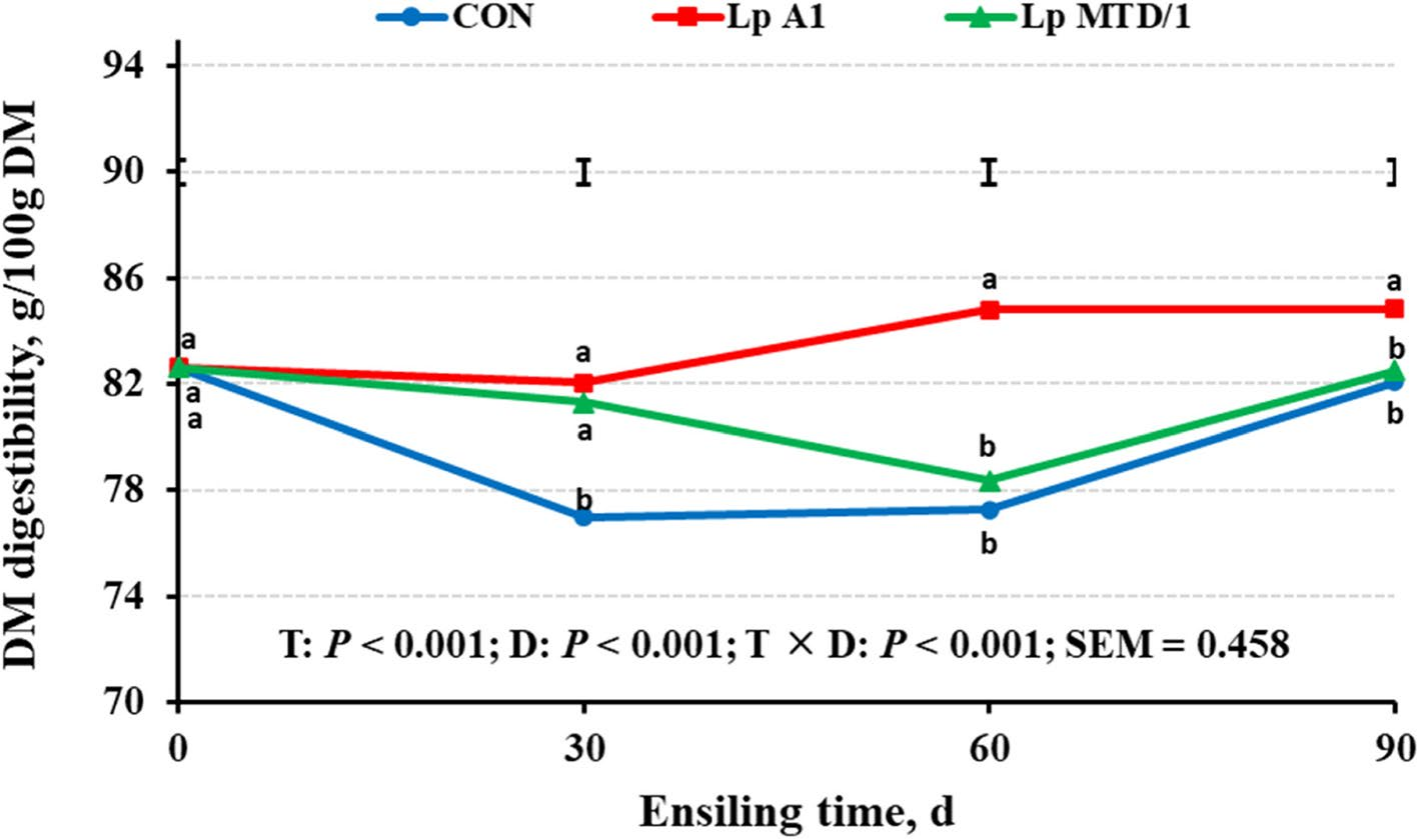
In vitro dry matter (DM) digestibility of alfalfa silages ensiled for 0 (fresh material), 30, 60 and 90 d. Treatments: CON = control (no additives); Lp A1 = alfalfa silage inoculated with *L. plantarum* A1; Lp MTD/1 = alfalfa silage inoculated with *L. plantarum* MTD/1. Means with different lowercase letters shows difference among treatments in the same ensiling day at *P* < 0.05 (*n* = 4, the number of replicates in each mean). T, effect of treatment; D, effect of ensiling time; T × D, interaction of treatment and ensiling time; bar and SEM indicate standard error of means (df = 47)

In order to investigate the effect of ensiling period on in vitro DM digestibility of silage, we measured the in vitro DM digestibility of silage samples at each ensiling stage using Daisy^II^ Incubator before the experiment. The results showed that alfalfa silages inoculated with Lp A1 and Lp MTD/1 had higher in vitro DM digestibility at 30 d of ensiling than CON, but by d 60 and 90, the highest DM digestibility was observed in Lp A1-treated silages, and a greater difference was found between Lp A1 and other treatments at d 60 (*P* < 0.05, Fig. 1). Thus, the 60^th^ d silage samples were used for further in vitro batch culture.

As expected, inoculation of Lp A1 in silage increased DM digestibility compared with CON and Lp MTD/1 treatments (Table 4; *P* < 0.05), but no difference was found between Lp A1 and FM treatments. Silage treated with Lp A1 had higher in vitro total gas production, total VFA, total branched-chain VFA and NH_3_-N than CON and Lp MTD/1 treatments (*P* < 0.05), while no difference was observed in these parameters between CON and Lp MTD/1 treatments.

**Table 4:** Effects of ferulic acid esterase-producing *L. plantarum* A1 inoculation in alfalfa silage on dry matter (DM) digestibility, gas production, and fermentation characteristics of in vitro fermentation for 48 h

Items^1^	Treatment^2^				SEM^3^	*P*-value
	FM	CON	Lp A1	Lp MTD/1		
DM degradability, %	69.9^ab^	67.6^bc^	72.0^a^	65.8^c^	0.78	0.003
Gas production						
Total gas, mL	187^a^	152^c^	168^b^	145^c^	4.99	< 0.001
Methane, mL	15.1^a^	14.1^b^	14.9^a^	12.7^c^	0.29	< 0.001
Methane, mL/g digestible DM	43.1^a^	41.7^a^	41.3^ab^	38.5^b^	0.57	0.007
Methane/Total gas, %	8.06^b^	9.26^a^	8.86^a^	8.73^a^	0.14	0.002
Volatile fatty acid (VFA)						
Total VFA, mmol/L	92.1^ab^	83.0^bc^	99.7^a^	77.4^c^	2.73	< 0.001
Acetate, mmol/L	58.2^a^	50.5^b^	60.9^a^	46.8^b^	1.82	< 0.001
Propionate, mmol/L	19.6^a^	16.9^b^	19.7^a^	15.2^b^	0.61	0.001
Acetate/Propionate	2.97^b^	3.04^a^	3.09^a^	3.07^a^	0.02	0.017
Butyrate, mmol/L	6.51^b^	6.50^b^	8.50^a^	6.34^b^	0.28	< 0.001
Valerate, mmol/L	3.21^c^	3.75^b^	4.37^a^	3.86^b^	0.13	< 0.001
Isobutyraye, mmol/L	1.42^c^	1.59^b^	1.92^a^	1.61^b^	0.06	< 0.001
Isovalerate, mmol/L	3.12^c^	3.83^b^	4.27^a^	3.52^b^	0.13	< 0.001
Total BCVFA, mmol/L	4.54^c^	5.42^b^	6.19^a^	5.12^b^	0.64	< 0.001
NH_3_-N, mmol/L	31.4^a^	25.5^b^	30.1^a^	25.9^b^	0.80	< 0.001

^a−c^Means within a row with different superscripts differ (*P* < 0.05)

^1^DM = dry matter; VFA = volatile fatty acid; BCVFA = branched-chain VFA; NH_3_-N = ammonia nitrogen

^2^FM = fresh material; CON = control (no additives); Lp A1 = silage inoculated with *L. plantarum* A1; Lp MTD/1 = silage inoculated with *L. plantarum* MTD/1

^3^SEM = standard error of means

**Fig. 2:**
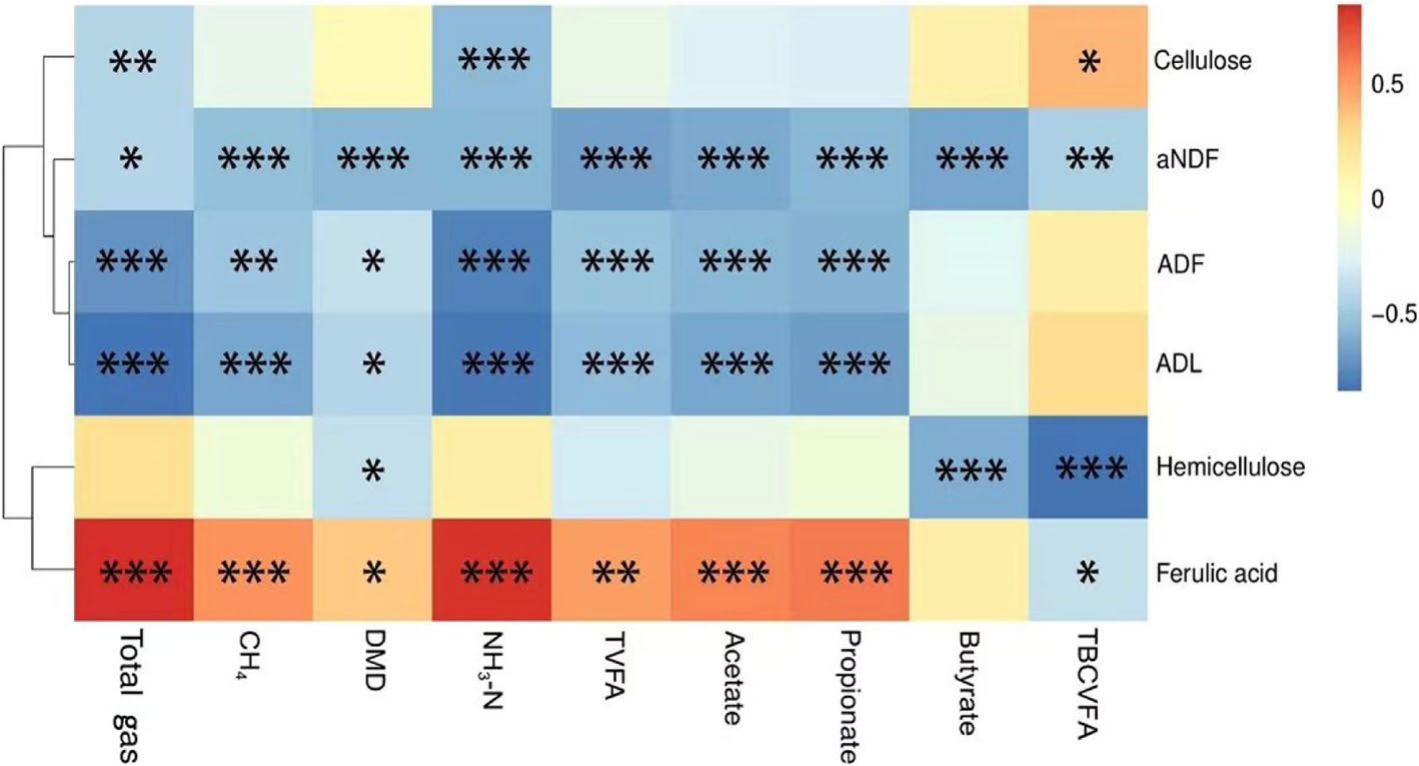
Correlations between the rumen fermentation parameters and silage chemical components. Cells are colored based on Pearson correlation coefficient (^*^P < 0.05; ^**^P < 0.01; ^***^P < 0.001)

The correlation of rumen fermentation indicators and silage fiber components was conducted using a correlation heatmap (Fig. 2). All detected rumen fermentation indicators were negatively correlated with aNDF ADF and ADL (*P* < 0.05), except for butyrate and total branched-chain VFA (BCVFA). The concentration of cellulose was negatively correlated with total gas production and NH_3_-N concentration, but positively correlated with total BCVFA. The concentration of hemicellulose was negatively correlated with DM digestibility, butyrate and total BCVFA concentrations.

### Effects of Lp A1 inoculation in silage on rumen microbial populations

**Fig. 3:**
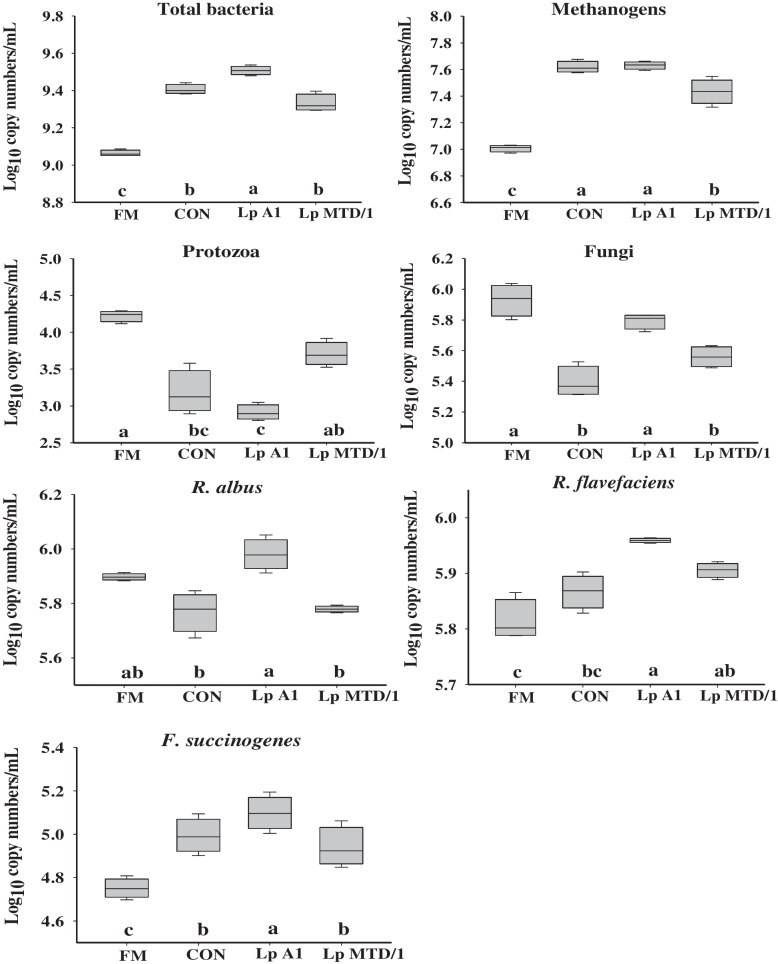
Effects of ferulic acid esterase-producing *L. plantarum* A1 inoculation in alfalfa silage on the population of total bacteria, protozoa, fungi, methanogens, *Ruminococcus albus*, *Ruminococcus flavefaciens* and *Fibrobacter succinogenes* (log_10_ copy number of the target genes/mL) in in vitro rumen cultures at 48 h. Treatment: FM = fresh material; CON = control (no additives); Lp A1 = silage inoculated with *L. plantarum* A1; Lp MTD/1 = silage inoculated with *L. plantarum* MTD/1. Means with different superscripts differ at *P* < 0.05

Quantitative real-time PCR showed that ensiling increased (*P* < 0.05) the populations of ruminal total bacteria, methanogens and *F. succinogenes*, but reduced the populations of protozoa and fungi in CON compared to FM (Fig. 3). Compared with CON, the populations of total bacteria, fungi, *R. albus* and *R. flavefaciens* were increased by Lp A1 (*P* < 0.05) inoculation but not by Lp MTD/1. The population of methanogens was reduced by Lp MTD/1 inoculation (*P* < 0.05) compared to that of CON, while no effect was observed in Lp A1 treatment. Both Lp A1 and Lp MTD/1 inoculations in silage didn’t affect the populations of ruminal protozoa and *F. succinogenes*.

### Change of rumen microbial community

The rumen microbial community was analyzed by Illumina sequencing of 16S rRNA gene. A total of 1,592,126 quality-checked sequences were generated as being bacterial across all 36 samples from the four treatments with average of 44,226 sequences for each sample were obtained. 99.96% of sequences had an average read length of 415 bp. Greater than 99% good’s coverage was achieved for all samples. The sequencing depth was also relatively comprehensive, as indicated by the rarefaction curve shown in Fig. S1.

**Fig. 4:**
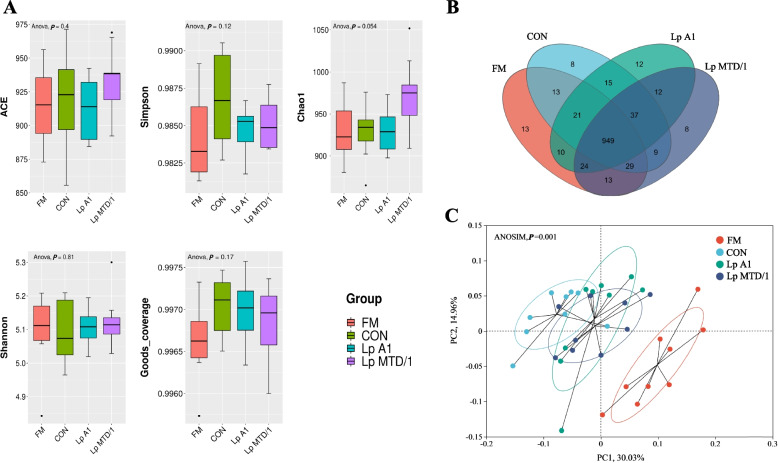
Bacterial community diversities of rumen fluid. **A** Alpha Diversity Index (including Shannon, Simpson, Chao 1, and ACE); **B** Venn diagram of ruminal microorganisms’ operational taxonomic unit (OTU); **C** Principal component analysis (PCoA) showing diversities of the bacterial communities in different treatments. Treatment: FM = fresh material; CON = control (no additives); Lp A1 = silage inoculated with *L. plantarum* A1; Lp MTD/1 = silage inoculated with *L. plantarum* MTD/1

The alpha diversity indices of the rumen microbial community were not influenced by ensiling or silage inoculants (*P* > 0.05; Fig. 4A). The Venn diagram showed the similarities and differences of ruminal microorganisms’ OUT detected in different treatment (Fig. 4B). A total of 949 OUT were simultaneously detected in 4 treatments. PCoA showed that microbial community structure tended to cluster based on the treatment (Fig. 4C). A clear separation was found between FM and silages along PC1, which explains > 30% of the total variation. In addition, the ruminal microbial communities of Lp A1 and Lp MTD/1 treatments were also separable with CON along PC1, while no distinction was noticed between Lp A1 and Lp MTD/1 treatments.

**Fig. 5:**
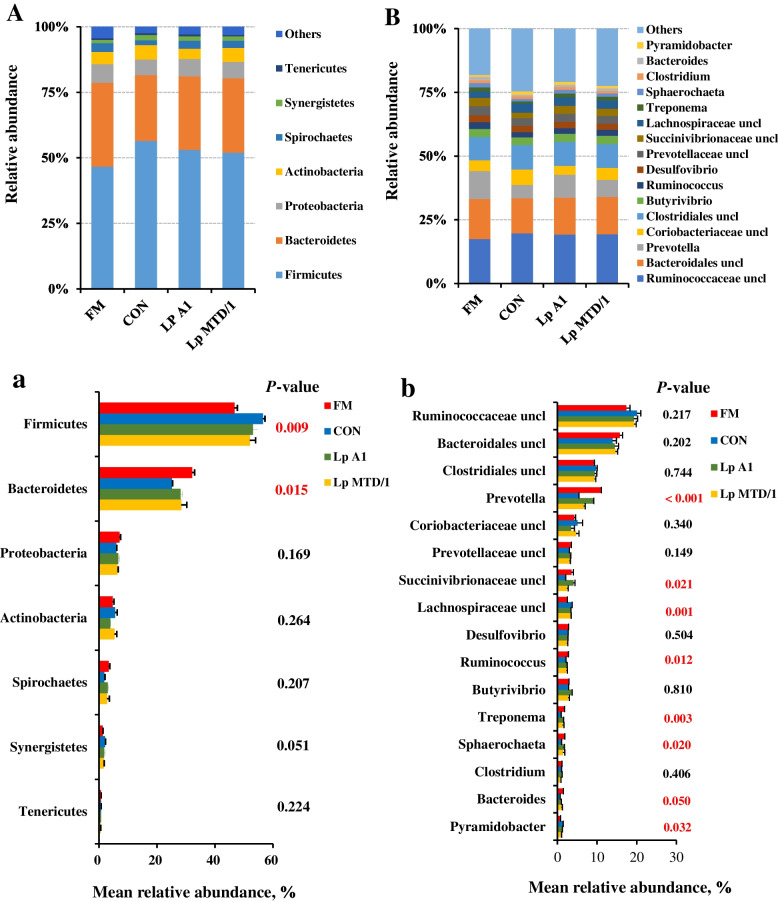
Effects of ferulic acid esterase-producing Lp A1 inoculation in silage on relative abundance of ruminal bacteria at phylum (**A** and **a**) and genus level (**B** and **b**) that accounted for ≥ 1% of total sequences in at least one treatment. Treatment: FM = fresh material; CON = control (no additives); Lp A1 = silage inoculated with *L. plantarum* A1; Lp MTD/1 = silage inoculated with *L. plantarum* MTD/1. uncl, unclassified

At the phylum level, a total of 7 bacteria phyla with an average relative abundance ≥ 1% were detected (Fig. 5A). Firmicutes (46.7%–56.5%) followed by Bacteroidetes (28.1%–32.1%) and Proteobacteria (5.91%–7.03%) were detected as the dominant bacterial phyla, together representing 85.7%–87.8% of all the sequences. Compared with FM, ensiling increased the relative abundance of Firmicutes in the culture of CON, but reduced the relative abundance of Bacteroidetes (*P* < 0.05). Both Lp A1 and Lp MTD/1 inoculated silages reduced the relative abundance of ruminal Firmicutes compared with CON, and a contrary result was observed in the relative abundance of Bacteroidetes (*P* < 0.05; Fig. 5a). Genus-level bacterial communities are shown in Fig. 5B and b. A total of 16 genera of bacteria were obtained to have a relative abundance greater than 1%, and these genera accounted for 75.1%–81.9% of the total sequences. Unclassified *Ruminococcaceae* (17.4%–20.0%) and unclassified *Bacteroidales* (13.9%–15.7%) were the most dominant bacteria. Although both Lp A1 and Lp MTD/1 inoculated silages increased the relative abundances of ruminal *Prevotella* and unclassified *Succinivibrionaceae* compared with CON, a greater effect was observed in Lp A1-treated group versus Lp MTD/1 treatment (*P* < 0.05). Besides, Lp A1- and Lp MTD/1-treated silages also had a parallel influence on some bacterial genera. The relative abundances of ruminal *Ruminococcus*, *Treponema*, *Sphaerochaeta* and *Bacteroides* were increased, while that of unclassified *Lachnospiraceae* and *Pyramidobacter* were decreased by Lp A1 and Lp MTD/1 inoculations.

### Correlations of ruminal bacterial communities with rumen fermentation characteristics

**Fig. 6:**
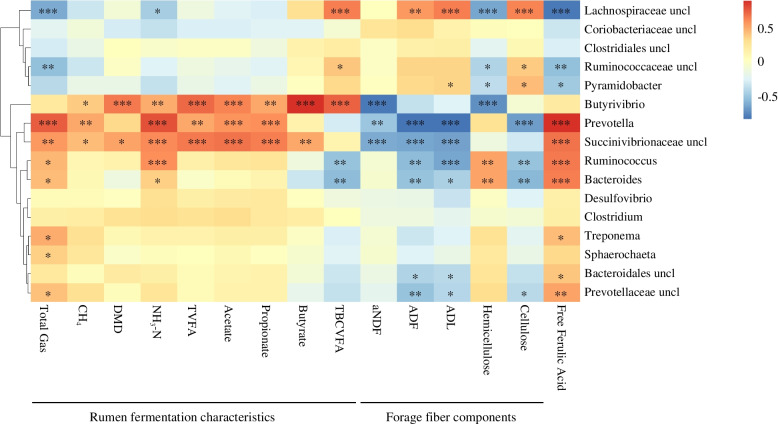
Correlations between the relative abundance of rumen bacteria (at genus level) and rumen fermentation parameters or silage chemical components. Cells are colored based on Pearson correlation coefficient (^*^P < 0.05; ^**^P < 0.01; ^***^P < 0.001). uncl, unclassified

Correlation analysis was conducted to evaluate the relationships of ruminal bacterial communities and rumen fermentation characteristics (Fig. 6). Total gas production was positively correlated with ruminal *Ruminococcus*, *Bacteroides*, *Prevotella*, unclassified *Prevotellaceae*, *Treponema*, *Sphaerochaeta* and unclassified *Succinivibrionaceae*, but negatively correlated with unclassified *Lachnospiraceae*, unclassified *Ruminococcaceae* and *Pyramidobacter*. Methane production, DM digestibility and VFA concentration were positively correlated with *Butyrivibrio*, *Prevotella* and unclassified *Succinivibrionaceae*. NH_3_-N was positively correlated with *Ruminococcus*, *Bacteroides*, *Prevotella*, *Butyrivibrio*, and unclassified *Succinivibrionaceae*, but negatively correlated with unclassified *Lachnospiraceae*.

## Discussion

### Conservation characteristics of alfalfa silage

Expectedly, inoculation with Lp A1 and Lp MTD/1 decreased silage pH as reported in a previous study [13], and the increases of lactic acid concentration in inoculated treatments account for the decrease of pH values [6, 18]. Compared with CON and Lp MTD/1-treated silages, inoculating Lp A1 in silage could produce FAE which acts as a biocatalytic agent to degrade lignocellulose structure during ensiling [14]. Afterwards, the water-soluble carbohydrates resulted from the lignocellulose degradation could be utilized by LAB to further promote fermentation [13, 33]. This was also confirmed by the substantial decrease in the fiber component and the increase of free ferulic acid concentration in Lp A1-treated silage. The results of DM loss further indicated that more nutrients were well preserved in Lp A1 and Lp MTD/1-treated silages compared with CON, which was consistent with previous studies [13, 18].

The Lp A1 is the major contributor to fiber degradation in silages of the present study due to its FAE producing ability. As previously reported, Lp A1 hydrolyzed the ester bond between lignin and structural polysaccharides by producing FAE during ensiling, thereby reducing the crystallinity and further enhancing the permeability of lignocellulose [10, 14]. Similar results had also been reported in our previous studies that lignocellulose was degraded in corn stalk, alfalfa and *Pennisetum sinese* silages when Lp A1 was used as an inoculant [12–14]. These results indicated that inoculation with both FAE-producing Lp A1 and the widely adopted commercial inoculant Lp MTD/1 in silage are effective treatments in improving alfalfa fermentation quality. But it is worth noting that Lp A1 also showed obvious fiber degradation performance at the same time compared with CON and Lp MTD/1-treated silage.

### Effects of Lp A1 inoculation in silage on the major microbial groups and bacterial community involved in feed digestion and methane production

Consistent with previous studies, inoculating FAE-producing Lp A1 in alfalfa silage increased alfalfa silage DM digestibility [15, 16, 34], resulting in the digestibility of DM very similar to that of the alfalfa before ensiling. This could be attributed to the ability of Lp A1 to preserve more digestible DM during silage fermentation, which in turn provided more substrate available for degradation by rumen microbiome [35]. In addition, the difference of the DM digestibility between Lp A1 and other treated silages may be related to the enzymolysis of the cross-link between arabinoxylans and lignin by the action of FAE produced by Lp A1 during ensiling, resulting in fiber to be more susceptible to attack by enzymes or rumen microorganisms [12, 14]. It can also be easily seen from the correlation heatmap that the digestion of silage is highly associated with the silage fiber components except for cellulose.

It has been proven that changes of forage fiber affect the rumen microbial community composition, which in turn affect fermentation products and ruminal pH [36, 37]. Therefore, increasing the knowledge of the rumen microbiomes and their transition in response to the alteration of feed’s fiber structure could help to improve the feed utilization efficiency of the host animal [37]. *R. albus*, *R. flavefaciens* and *F. succinogenes* are considered the predominant rumen fibrolytic bacteria due to their high cellulose digestion ability [29, 38]. Moreover, protozoa and fungi have also been reported to take function in fiber degradation [29, 37, 39]. Consistent with previous findings, we detected more total bacteria, *R. albus*, *R. flavefaciens* and fungi in Lp A1 treatment, compared with CON and Lp MTD/1 treated group [40–42]. Thus, the increased total bacteria, *R. albus*, *R. flavefaciens* and fungi might have resulted in the improved silage DM digestibility in the Lp A1 treatment [29, 42]. In addition, based on 16S rRNA sequencing analysis, the main changes in the bacterial community as a result of Lp A1 inoculation were significant increases in the abundances of *Prevotella*, *Butyrivibrio* and unclassified *Succinivibrionaceae*. *Butyrivibrio* is recognized as fibrolytic bacterial genera which was increased in Lp A1 treatment [29, 43]. As a core genus in the rumen, *Prevotella* was also increased in Lp A1 treatment, and the relative abundance was similar to that of FM. Studies have shown that *Prevotella* app. are highly active hemicellulolytic and proteolytic bacteria [42, 44]. Furthermore, ruminal *Prevotella* is also known to actively take part in the breakdown of starch, xylan and pectin during digestion and utilization [44, 45]. The increase in these bacteria is also crucial for the efficient degradation and utilization of cellulosic and non-cellulosic plant polysaccharide and protein in the rumen.

The results of the present study indicated that the silage inoculation with Lp A1 increased DM digestibility with a concomitant increase in methanogenesis, which is consistent with the notion that the higher methane production could be due to the higher in vitro DM digestibility [46]. Thus, this finding largely associates with the increased amounts of fermentable substrates in the in vitro fermenters [47]. However, it is worth noting that the methane production in Lp A1 treatment is similar to that of the CON on the basis of per gram of digestible DM. On the contrary, methane production was reduced by Lp MTD/1 inoculation in silage without affecting the in vitro DM digestibility, which was consistent with previous studies [35, 48, 49]. The rumen microbial mechanism of methanogenesis from Lp A1 and Lp MTD/1 inoculated silages might be different. In the current study, silages inoculated with Lp A1 had no effect on the population of methanogens, but it had been reduced in Lp MTD/1 treated group. Muck et al. [50] reported that some inoculant-treated silages reduced gas production compared with the untreated silage, suggesting that a modification had occurred in the ruminal fermentation process. But the specific reason for the inhibition of methanogens is not clearly known, which require further investigation for verification.

### Effects of Lp A1 inoculation in silage on the major microbial groups and bacterial community involved in rumen fermentation

Acetate, propionate and butyrate are the key VFA produced in the rumen from the fermentation of dietary carbohydrates, and these could provide ruminants with up to 70%–80% of all their energy requirements [51]. The greater total VFA concentration in the rumen generally resulted from the higher DM degradation of the diet [2, 35], which supplied more fermentation substrate for rumen microbes. In the current study, silage inoculated with Lp A1 increased the ruminal acetate, propionate, and butyrate concentrations after 48 h of in vitro incubation, but it did not affect the ratio of acetate to propionate. The increased VFA concentration in Lp A1 treatment was probably related to the increase of Gram-positive fibrolytic bacteria belonging to *Ruminococcus* spp. [29, 52, 53]. Therefore, we posited that more structural carbohydrates were disintegrated to hexoses or pentoses, which were then rapidly converted to pyruvate and finally to acetate, propionate, and butyrate in Lp A1 treatment [35].

The cleavage of the acetyl linkages during the hydrolysis of the hemicellulose may have actuated the removal of acetyl groups, resulting in the formation of more free acetate in the culture [14]. An unsubstantiated proposition by Shen et al. [29] suggested that some Gram-positive bacteria, such as those unclassified bacteria in *Ruminococcaceae* and *Lachnospiraceae* were also positively correlated with acetate concentration. However, our results did not concur with the above proposition. *Prevotella* is a genus consisting of proteolytic, amylolytic, and hemicellulolytic bacteria, dominating the rumen of adult ruminants and producing succinate and acetate [44, 54]. Therefore, the higher abundance of *Prevotella* in Lp A1 treatment might be another reason for the increased acetate and propionate concentration. The present results suggested that the production of acetate was basically related to the fiber degrading bacteria of the rumen, while the efficiency of fiber-degrading bacteria was facilitated by the change in the fiber structure during silage fermentation.

Propionate is produced via succinate (randomizing pathway) or acrylate (non-randomizing pathway) in the rumen, and the succinate pathway is regarded as the major pathway [29, 55]. In addition to *Prevotella*, which fermentation products include succinate [44, 56], previous studies have also shown that fermentation end-products of *F*. *succinogenes* and *R. flavefaciens* are mainly succinate, which could be used for propionate production in the rumen [52, 55]. In the current study, silage inoculated with Lp A1 did not affect the population of ruminal *F*. *succinogenes* compared with CON and Lp MTD/1 treatments. Thus, a possible reason for the higher propionate concentration in Lp A1 treatment is that the population of *R. flavefaciens* was greater in this group. Consistent with previous studies, higher abundance of unclassified *Succinivibrionaceae* had been found in the rumen culture of Lp A1 treatment, indicating that this taxon might also play an important role in the increased propionate production [56, 57]. Besides, it's worth noting that the higher propionate concentration in Lp A1 treatment probably associated with the high lactic acid concentration of the silage, since lactate could be converted to propionate in the rumen by lactate-utilizing bacteria such as *Megasphaera elsdenii* and *Selenomonas ruminantium* [52].

*Butyrivibrio* and *Pseudobutyrivibrio* are regarded as important butyrate-producing genera in the rumen [29], but the relative abundances of *Pseudobutyrivibrio* was not different among treatments. Additionally, *Pseudobutyrivibrio* was not considered due to the abundance of *Pseudobutyrivibrio* (< 0.1%) was much less than that of *Butyrivibrio*. Hence, based on the positive correlations between *Butyrivibrio* and butyrate concentration, we speculated that the high abundance of *Butyrivibrio* in the Lp A1 treatment caused the higher butyrate production. Valerate and branched-chain VFA (i.e., isovalerate and isobutyrate) in the rumen primarily originate from ruminal oxidative-deamination and decarboxylation of valine, leucine, and isoleucine [36, 58]. Therefore, the increased valerate and branched-VFA concentration in Lp A1 treated group may result from increased amino acid deamination by microbes if the utilization rate was not changed. Even though we did not determine the main hyper-ammonia-producing bacteria in our study, it could be substantiated by the higher ammonia concentration in the Lp A1 treatment. The higher concentration of ammonia in FM group might attribute to the low total bacterial populations that reduced ammonia utilization by microbes [29].

## Conclusions

Inoculating alfalfa silage with either Lp A1 or Lp MTD/1 improved the fermentation quality. However, Lp A1 exerted greater effects on fiber degradation as indicated by the low fiber content and high free ferulic acid concentration of the treated silages. Noteworthily, alfalfa silage inoculated with Lp A1 improved in vitro DM digestibility than CON and Lp MTD/1 treatments. Additionally, although inoculating alfalfa silage with Lp A1 had little effect on the rumen’s microbial alpha diversity, it had triggered different modifications of the rumen microbiota resulting in superior rumen fermentation characteristics. Therefore, our findings suggest that alfalfa silage inoculated with FAE-producing Lp A1 could be practically more effective in improving silage quality and digestibility as well as modulating the rumen fermentation which ultimately translate to efficient feed utilization. Our results provide an important basis for deeper understandings and further research on practical application of FAE-producing LAB as silage inoculants.

## Supplementary Information

**Additional file 1:** **Table S1.** Primers and corresponding amplification conditions used for quantitative real-time PCR in this study.

**Additional file 2:** **Fig. S1.** Rarefaction of four treatments. Treatment: FM = fresh material; CON = control (no additives); Lp A1 = silage inoculated with *L. plantarum* A1; Lp MTD/1 = silage inoculated with *L. plantarum* MTD/1.

## Data Availability

The datasets used and/or analyzed during the current study are available from the corresponding author on reasonable request. Raw sequencing files have been deposited in the NCBI database (https://www.ncbi.nlm.nih.gov/sra/PRJNA793346).

## Abbreviations

ADF: Acid detergent fiber
ADL: Acid detergent lignin
BCVFA: Branched-chain volatile fatty acid
CP: Crude protein
CON: Control (no additives)
DM: Dry matter
FAE: Ferulic acid esterase
FM: Fresh material
LAB: Lactic acid bacteria
Lp A1: Silage inoculated with *Lactiplantibacillus plantarum* A1
Lp MTD/1: Silage inoculated with *Lactiplantibacillus plantarum* MTD/1
NDF: Neutral detergent fiber
NH_3_-N: Ammonia nitrogen
OUT: Operational taxonomic units
PcoA: Principal coordinate analysis
PCR: Polymerase chain reaction
qPCR: Quantitative real-time PCR
VFA: Volatile fatty acid
WSC: Water soluble carbohydrates
