# Correction: Effects of inoculating feruloyl esterase-producing Lactiplantibacillus plantarum A1 on ensiling characteristics, in vitro ruminal fermentation and microbiota of alfalfa silage

**DOI:** 10.1186/s40104-023-00871-y

**Published:** 2023-04-07

**Authors:** Fuhou Li, Samaila Usman, Wenkang Huang, Mengya Jia, Zohreh Akhavan Kharazian, Tao Ran, Fadi Li, Zitong Ding, Xusheng Guo

**Affiliations:** 1 State Key Laboratory of Herbage Improvement and Grassland Agro-Ecosystems, College of Pastoral Agriculture Science and Technology, Lanzhou University, Lanzhou, 730000 People’s Republic of China; 2 Probiotics and Biological Feed Research Centre, Lanzhou University, Lanzhou, 730000 People’s Republic of China; 3 State Key Laboratory of Herbage Improvement and Grassland Agro-Ecosystems, School of Life Sciences, Lanzhou University, No. 222 South Tianshui Road, Lanzhou, 730000 People’s Republic of China

**Correction: J Animal Sci Biotechnol 14, 43 (2023)**

**https://doi.org/10.1186/s40104-023-00837-0**

After publication of the original article [1], it was reported that there were four words missing in section ‘Chemical analysis of fresh alfalfa and silage’.

On page 3, in the right column, the words “[amylase-treated neutral detergent fiber (ADF), acid detergent lignin (ADL)]” was incomplete.

The correct texts should read: [amylase-treated neutral detergent fiber (aNDF), acid detergent fiber (ADF), acid detergent lignin (ADL)].

The original article [1] has been updated.
