# Supplementary material for: Effects of inoculating feruloyl esterase-producing Lactiplantibacillus plantarum A1 on ensiling characteristics, in vitro ruminal fermentation and microbiota of alfalfa silage
Source: J Anim Sci Biotechnol. 2023 Mar 14;14:43. doi: 10.1186/s40104-023-00837-0 (PMC10012570; doi:10.1186/s40104-023-00837-0)
Supplement: Supplementary file 1 — Additional file 1: Table S1. Primers and corresponding amplification conditions used for quantitative real-time PCR in this study. [file 40104_2023_837_MOESM1_ESM.docx]

**Table S1** Primers and corresponding amplification conditions used for quantitative real-time PCR in this study

| **Target species** | **Primer** | **Sequence（5’** **→3’）** | **Siz， bp** | **Thermal profile** |
| --- | --- | --- | --- | --- |
| Total bacteria | Total bacteria-F [1]  Total bacteria-R [1] | CGGCAACGAGCGCAACCC | 147 | 95 ℃ for 3 min × 1 cycle；95 ℃ for 30 s，56 ℃ for 30 s，72℃ for 40 s × 35cycles； |
|  |  | CCATTGTAGCACGTGTGTAGCC |  |  |
| Protozoa | Protozoa-F [1] | GCTTTCGWTGGTAGTGTATT | 234 | 95 ℃ for 3 min × 1 cycle；95 ℃ for 30 s，60 ℃ for 30 s，72 ℃ for 40 s × 35cycles； |
|  | Protozoa-R [1] | CTTGCCCTCYAATCGTWCT |  |  |
| Fungi | Fungi-F [2] | GAGGAAGTAAAAGTCGTAACAAGGTTTC | 120 | 95 ℃ for 3 min × 1 cycle；95 ℃ for 30 s，60 ℃ for 30 s，72 ℃ for 40 s × 35 cycles； |
|  | Fungi-R [2] | CAAATTCACAAAGGGTAGGATGATT |  |  |
| Methanogens | Methanogen-F [1] | TTCGGTGGATCDCARAGRGC | 160 | 95 ℃ for 3 min × 1 cycle；95 ℃ for 30 s，56 ℃ for 30 s，72 ℃ for 40 s × 35 cycles； |
|  | Methanogen-R [1] | GBARGTCGWAWCCGTAGAATCC |  |  |
| *Ruminococcus albus* | *Ruminococcus albus*-F [1] | CCCTAAAAGCAGTCTTAGTTCG | 176 | 95 ℃ for 3 min × 1 cycle；95 ℃ for 30 s，56 ℃ for 30 s，72 ℃ for 40 s × 35 cycles； |
|  | *Ruminococcus albus*-R [1] | CCTCCTTGCGGTTAGAACA |  |  |
| *Ruminococcus flavefaciens* | *Ruminococcus flavefaciens*-F [2] | CGAACGGAGATAATTTGAGTTTACTTAGG | 131 | 95 ℃ for 3 min × 1 cycle；95 ℃ for 30 s，56 ℃ for 30 s，72 ℃ for 40 s × 35 cycles； |
|  | *Ruminococcus flavefaciens*-R [2] | CGGTCTCTGTATGTTATGAGGTATTACC |  |  |
| *Fibrobacter succinogenes* | *Fibrobacter succinogenes*-F [1] | GTTCGGAATTACTGGGCGTAAA | 121 | 95 ℃ for 3 min × 1 cycle；95 ℃ for 30 s，60 ℃ for 30 s，72 ℃ for 40 s×35 cycles |
|  | *Fibrobacter succinogenes*-R [1] | CGCCTGCCCCTGAACTATC |  |  |

**Reference:**

1. Iqbal MW, Zhang Q, Yang Y, Zou C, Li L, Liang X, et al. Ruminal fermentation and microbial community differently influenced by four typical subtropical forages in vitro. Anim Nutr. 2018;4:100–8. https://doi.org/10.1016/j.aninu.2017.10.005.
2. Denman SE, McSweeney CS. Development of a real-time PCR assay for monitoring anaerobic fungal and cellulolytic bacterial populations within the rumen. FEMS Microbiol Ecol. 2006;58:572–82. https://doi.org/10.1111/j.1574-6941.2006.00190.x.
