# Supplementary material for: Effects of inoculating feruloyl esterase-producing Lactiplantibacillus plantarum A1 on ensiling characteristics, in vitro ruminal fermentation and microbiota of alfalfa silage
Source: J Anim Sci Biotechnol. 2023 Mar 14;14:43. doi: 10.1186/s40104-023-00837-0 (PMC10012570; doi:10.1186/s40104-023-00837-0)
Supplement: Supplementary file 2 — Additional file 2: Fig. S1. Rarefaction of four treatments. Treatment: FM = fresh material; CON = control (no additives); Lp A1 = silage inoculated with L. plantarum A1; Lp MTD/1 = silage inoculated with L. plantarum MTD/1. [file 40104_2023_837_MOESM2_ESM.docx]

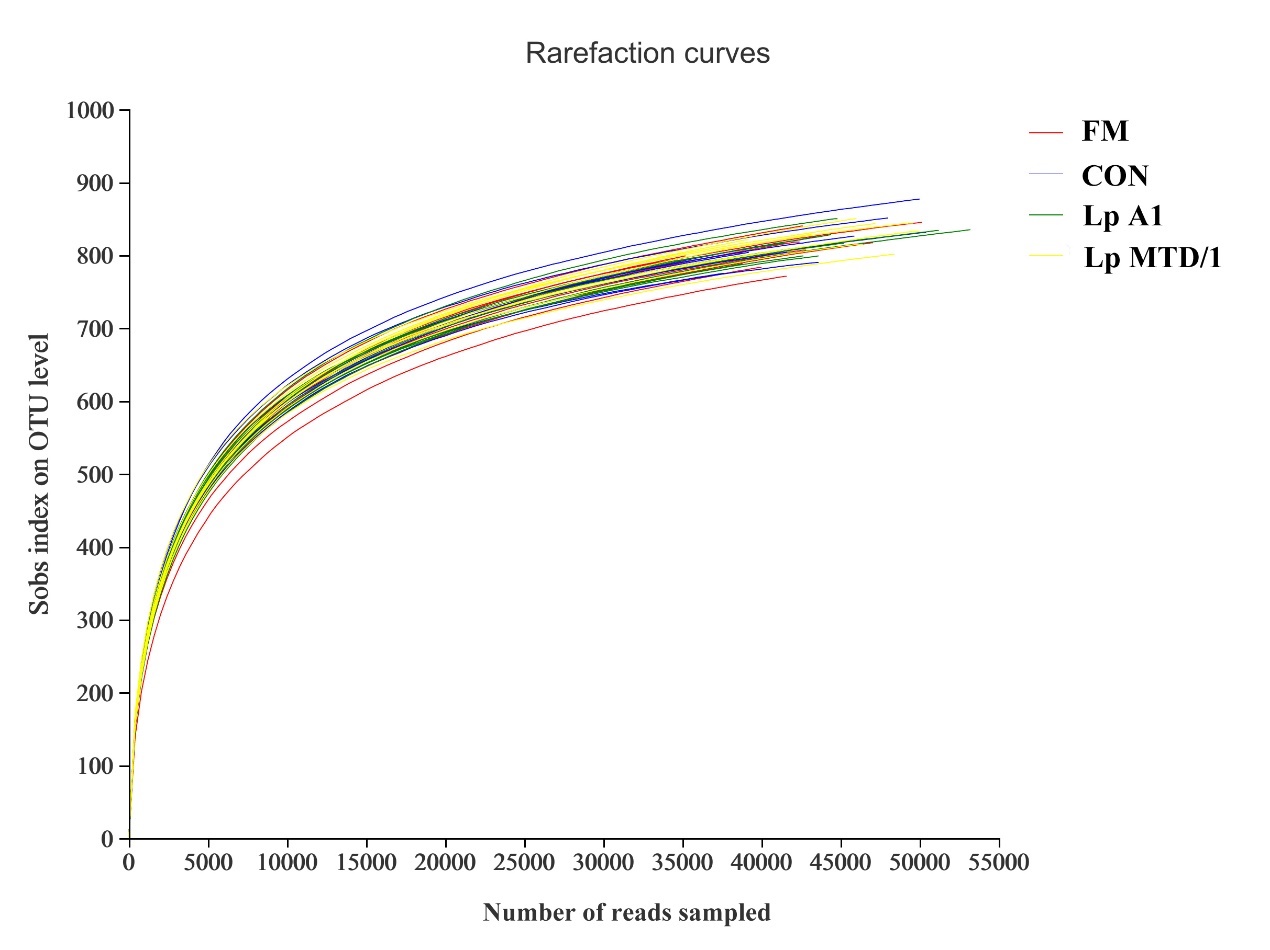


**Fig. S1.** Rarefaction of four treatments. Treatment: FM = fresh material; CON = control (no additives); Lp A1 = silage inoculated with *L. plantarum* A1; Lp MTD/1 = silage inoculated with *L. plantarum* MTD/1
